# Highly Efficient Aggregation-Induced Room-Temperature Phosphorescence with Extremely Large Stokes Shift Emitted from Trinuclear Gold(I) Complex Crystals

**DOI:** 10.3390/molecules24244606

**Published:** 2019-12-16

**Authors:** Osamu Tsutsumi, Masakazu Tamaru, Hitoya Nakasato, Shingo Shimai, Supattra Panthai, Yuki Kuroda, Kenta Yamaguchi, Kaori Fujisawa, Kyohei Hisano

**Affiliations:** Department of Applied Chemistry, Ritsumeikan University, 1–1–1 Nojihigashi, Kusatsu 525-8577, Japan; konbudashi22@gmail.com (M.T.); 27111000455nz@gmail.com (H.N.); shingo1220535@gmail.com (S.S.); gr0344hv@ed.ritsumei.ac.jp (S.P.); sc0050ir@ed.ritsumei.ac.jp (Y.K.); sc0075vi@ed.ritsumei.ac.jp (K.Y.); kfujisawa@kyulux.com (K.F.); hisano@fc.ritsumei.ac.jp (K.H.)

**Keywords:** Au complex, aggregation-induced emission, phosphorescence, Stokes shift

## Abstract

Highly efficient (≈75% quantum yield), aggregation-induced phosphorescence is reported. The phosphorescence is emitted at room temperature and in the presence of air from crystals of trinuclear Au(I) complexes, accompanied by an extremely large Stokes shift of 2.2 × 10^4^ cm^−1^ (450 nm). The mechanism of the aggregation-induced room-temperature phosphorescence from the Au complex crystals was investigated in terms of the crystal packing structure and the primary structure of the molecules. It was found that two kinds of intermolecular interactions occurred in the crystals, and that these multiple dual-mode intermolecular interactions in the crystals play a crucial role in the in-air room-temperature phosphorescence of the trinuclear Au(I) complexes.

## 1. Introduction

Aggregation-induced emission (AIE) is a fascinating phenomenon wherein molecules display intense luminescence by aggregation [1,2,3]. Organic molecules can exhibit efficient luminescence in dilute solutions, but their luminescence efficiency is decreased by aggregation in condensed phases by aggregation-caused quenching (ACQ), a well-known phenomenon which is very common among organic luminescent molecules [3,4,5,6]. However, the development of practical applications using luminescent molecules is hindered by the ACQ of conventional luminogens; and attaining intensive photoluminescence in condensed phases or aggregates is a vital parameter for improving their usefulness. To address the recent demand for efficient luminescence materials in molecular aggregates, AIE-active luminogens (AIEgens) have been developed, and have attracted prime interest as practical luminescent materials for biological imaging, chemical sensing, optoelectronic systems, and related applications [7,8,9,10,11,12]. A general working mechanism of the AIE involves restriction of intramolecular motions that lead to non-radiative deactivation of excited states, such as vibration and rotation, due to aggregation [1,2,3]. Great effort has been devoted to further restricting intramolecular motions using the intermolecular interactions of AIEgens.

Molecules containing Au(I) ions are examples of AIEgens that exhibit strong photoluminescence in condensed phases [13,14,15,16,17]. The aurophilic interaction between Au atoms in Au containing complexes is an important property which induces luminescence in their aggregates [13,14]. As luminescence results from the Au–Au interaction, Au complexes exhibit unique luminescence in their condensed phase, and their luminescence properties, such as intensity or color, depend on the Au–Au distance, which can be tuned by modifying the structure of their aggregates [18,19,20,21,22,23,24,25,26,27,28,29,30,31]. Thus far, modifications to structures of molecular aggregates in the solids by external stimuli have been reported for modifying the photoluminescence properties of Au complexes [8,9,10,11]. For instance, in Au complexes exhibiting polymorphism, the thermal phase transition causes a drastic change in the luminescence color and intensity [26,27,28,29,30,31].

**Scheme 1:**
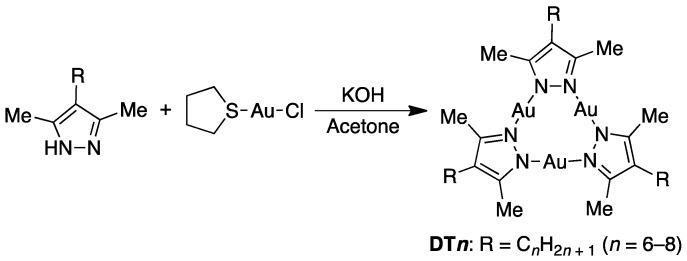
Synthesis and molecular structure of the trinuclear Au(I) complexes **DT*n***.

In this study, we designed trinuclear Au complexes represented by **DT*n*** (*n* = 6–8) to induce multiple Au–Au interactions (Scheme 1) [32,33,34], and investigated their luminescence behaviors in solids. We hypothesized that multiple interactions could enhance AIE activity, and that very efficient AIE could be obtained from trinuclear Au complex solid materials [19,20,21]. The stabilization energy of the Au–Au interaction is as large as that of the hydrogen bond [13,14,15,16], and for this reason it is often utilized for the formation of supramolecular assemblies [18,19,20,21]. We also anticipated that multiple Au–Au intermolecular interactions would improve the restriction of the internal motion and would further enhance the AIE effects in condensed phases of the complexes. Previously, the luminescence behavior of **DT6** (Scheme 1) in a poly(methyl methacrylate) (PMMA) dispersion was reported; the complex showed efficient phosphorescence in PMMA films [34]. In this study, the mechanism of the efficient phosphorescence of the trinuclear complexes **DT*n*** is discussed in relation to the structures of the molecular aggregates. Therefore, the luminescence behaviors of the complexes were observed in crystal, in where the molecular aggregated structure is well defined, to understand the relationship between aggregated structure and photophysical properties. Here, we report that the complex crystals emitted highly efficient room-temperature phosphorescence (RTP) in the presence of air, accompanied by an extremely large Stokes shift. The mechanism of the in-air RTP from the crystals of complexes is discussed in terms of the structures of the molecular aggregates in the crystals.

## 2. Results and Discussion

### 2.1. Synthesis and Characterization of Complexes

The molecular structure and synthesis of **DT*n*** are shown in Scheme 1. Our molecular design strategy centered around the use of a simple ligand without bulky substituents around the Au atoms to improve the Au–Au intermolecular interaction, which is typically blocked by the steric hindrance of bulky ligands. Therefore, a simple pyrazole derivative with a long n-alkyl side chain at the 4-position was designed as the ligand for the trinuclear Au complexes. The pyrazole ligands were synthesized from commercially available 2,4-pentanedione in two steps. The trinuclear Au complexes **DT*n*** were then prepared by complexation of (tht)AuCl (tht: tetrahydrothiophene) and the corresponding pyrazole ligands [32,33,34]. All the synthesized complexes were purified by silica gel column chromatography and recrystallisation. The complexes were fully characterized, and the analytical data confirmed that the desired complexes were obtained in high purity. The related analytical data are provided in the experimental section. Details of the preparation of the pyrazole ligands are provided in the Electronic Supplementary Information (ESI) and ^1^H and ^13^C-NMR spectra of the final compounds are shown in Figures S1–S3.

### 2.2. Photoluminescence Behavior of the Complexes

**Figure 1:**
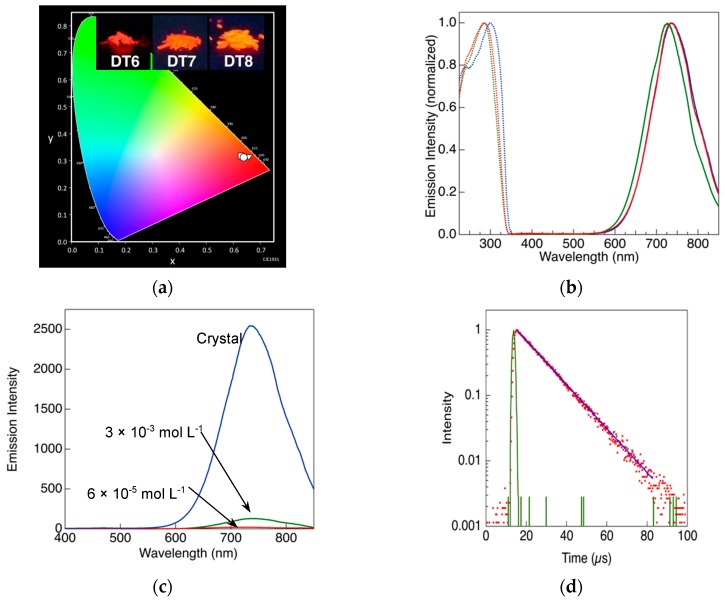
(**a**) Photograph of crystals taken under UV irradiation at 254 nm using a handheld lamp, and CIE (Commission Internationale de l’Éclairage) chromaticity diagram of the photoluminescence of the crystals: ●, **DT6**; ▼, **DT7**; ■, **DT8**. (**b**) Photoluminescence (solid line) and excitation spectra (dotted line) of the crystals of **DT*n*** at room temperature in air (red: **DT6**, green: **DT7**, blue: **DT8**). The excitation wavelength (*λ*_ex_) was 280 nm for the luminescence spectra, and the luminescence was monitored at their *λ*_max_^lum^ for the excitation spectra (Table 1). (**c**) Photoluminescence spectra of **DT6** in crystalline form and in hexane solutions (*λ*_ex_ = 280 nm). Concentrations of the hexane solutions are indicated in the Figure. (**d**) Decay profile of the luminescence of the **DT6** crystal at 733 nm at ambient temperature (*λ*_ex_ = 330 nm; pulse width = 600 ps): red, observed luminescence decay; blue, fitting curve; green, instrument response function.

The recrystallized complexes were placed between a pair of quartz plates and irradiated with UV light at 254 nm at ambient temperature in the presence of air. Bright red photoluminescence could be observed from all complexes using the naked eye (Figure 1a). The emission and excitation spectra of the Au complex crystals at ambient temperature are shown in Figure 1b, and the quantitative photophysical parameters for the room-temperature photoluminescence of the crystals are summarized in Table 1. The crystals of all the complexes exhibited broad unstructured luminescence at around 730 nm with significantly high quantum yields (*Φ*). The highest *Φ* was obtained for the **DT6** crystal; *Φ* was 75% for **DT6**, while it was ~60% for the other complexes. In contrast, we could not observe such intense luminescence in dilute solutions of any of the complexes. Figure 1c shows the luminescence spectra of **DT6** in hexane solutions as a representative example. A 10^−5^ mol L^−1^ solution of **DT6** exhibited no luminescence, and only very weak luminescence was observed at the same wavelength as in the crystal in a concentrated solution (10^−3^ mol L^−1^). Hexane is actually a poor solvent for these complexes, and it can be assumed that aggregates of the complexes are formed in concentrated hexane solutions. These results indicate that the trinuclear Au complexes we prepared cannot emit luminescence in dilute solution, wherein only isolated molecules exist, which suggests that the **DT*n*** complexes are AIE active and that the luminescence of the complexes is induced by the formation of aggregates. The AIE activity was further confirmed through the observation of luminescence behavior in the mixed solvent system for **DT6** (Figure S7d). In pure CH_2_Cl_2_, which is a good solvent for **DT6**, the complex showed no luminescence; however, the addition of methanol, which is a poor solvent, to the solution with the volume fraction of >80% to induce the aggregation of molecules, significantly enhanced the emission (Figure S7e).

Notably, the complexes displayed extremely large Stokes shifts. The absorption maxima of the complexes in dilute solutions were around 250 nm (Figure S6), and the absorption maxima of the crystals, estimated from the excitation spectra (Figure 1b) were located at 280 nm. Therefore, the crystals displayed a Stokes shift of at least 2.2 × 10^4^ cm^−1^ (450 nm) in all complexes. This feature is highly favorable for their application as imaging and sensing materials [1,2,3].

To obtain more information about the photoluminescence properties of the complexes, we measured the luminescence lifetime (*τ*) of their crystals at the wavelength of their luminescence maxima (*λ*_max_^lum^). The luminescence decay profile for the **DT6** crystal is shown in Figure 1d as a representative example. The crystals of all the complexes exhibited a single-exponential decay profile at room temperature (Figure 1d and Figure S9). The *τ* values determined from the decay profile were in the microsecond range for all the complexes, indicating that the observed luminescence of the crystals at room temperature was phosphorescence. Although the luminescence was phosphorescence, the crystals of the prepared complexes exhibited very high *Φ* values at room temperature in the presence of air (*Φ* = 61%–75%). It is well-known that the triplet excited state is easily quenched by the molecular oxygen in air. In addition, the rate constants for phosphorescence emission (*k*_r_) are much smaller than those of non-radiative deactivation processes (*k*_nr_) at ambient temperature, as the T_1_–S_0_ radiative transition is a spin-forbidden process. Therefore, although it has been reported that some specific organic and organometallic materials exhibit RTP recently [1,2,3], phosphorescence is typically observed only at low temperatures in the absence of oxygen molecules. Considering such sensitivity, it is noteworthy that the complexes reported here exhibited high *Φ* values under room-temperature conditions in the presence of air.

### 2.3. Relationship between the Room-Temperature Phosphorescence Properties and Aggregated Structure

**Figure 2:**
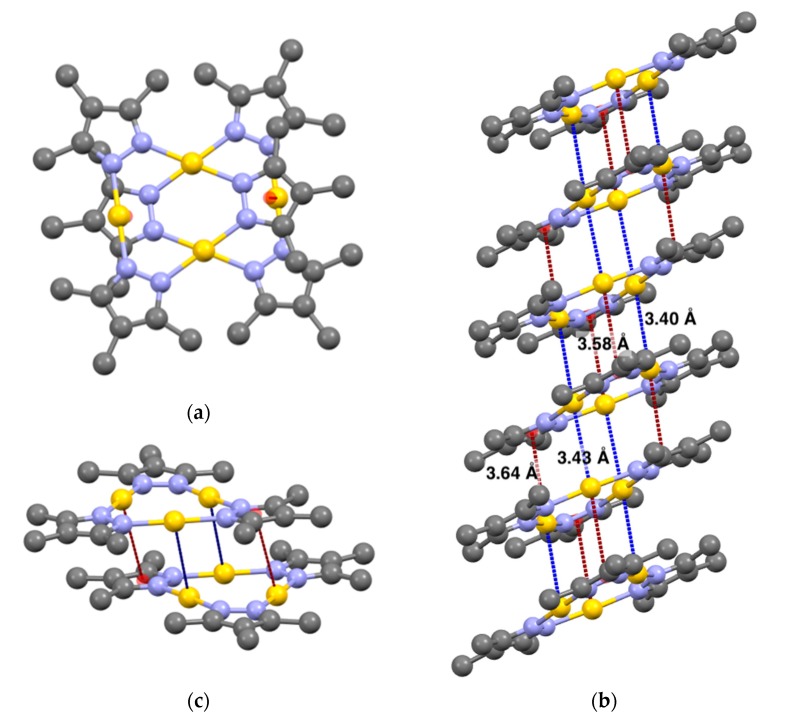
Crystal structure of **DT6** at room temperature. (**a**,**c**) Structure of the dimer: (**a**) top view; (**c**) side view. (**b**) Structure of the column-like supramolecular polymer; three dimers are extracted from the polymer. For clarity, H atoms and alkyl side chains are omitted. Atom color legend: grey, C; purple, N; yellow, Au; red, the centroid of pyrazole ring. Intermolecular Au–Au and Au–π interactions are indicated using blue and red lines, respectively.

To understand the underlying reason for the efficient in-air RTP of the **DT*n*** crystals, we analyzed their crystal structures. Single crystals of all the complexes were obtained by slow evaporation from a mixed solvent system of dichloromethane and acetone, and analyses of their single-crystal X-ray structures were performed. Their crystal packing structures are shown in Figure 2, Figures S4 and S5, in which two neighboring molecules are extracted and shown to discuss their intermolecular interactions in the crystals. The related key crystallographic data is listed in Table S1. Select interatomic distances between neighboring complexes are summarized in Table 2 to analyze the structure–property relationships of the complexes. The X-ray structure analysis of **DT*n*** indicated that the intermolecular Au–Au distance was 3.40 Å or less. It is generally accepted that the Au–Au distance is in the range of 2.50–3.50 Å, substantially shorter than the sum of the van der Waals radii (3.8 Å) of two Au atoms, suggesting the existence of an aurophilic interaction between them [16]. Furthermore, the Au atom was located close to the pyrazole ring of the neighboring molecule; the distance between the Au atom and centroid of the pyrazole ring was determined to be 3.58 Å or less, and the angle between the vector normal to the pyrazole ring and that passing through the centroid of the Au atom (*θ*) was 2.4°–15°. Au complexes form aggregates due to the interactions between the Au atoms and aromatic π-electron systems (Au–π interaction) when the distance between them is less than 4.0 Å, with the *θ* angle being equal to or less than 20° [35]. Therefore, the geometric parameters obtained from the single-crystal structural analysis of **DT*n*** indicated the presence of an intermolecular Au–π interaction between neighboring molecules. The analysis further indicated that all the complexes formed dimers in the crystal via both non-covalent Au–Au and Au–π intermolecular interactions (Figure 2c, Figures S4 and S5).

**Figure 3:**
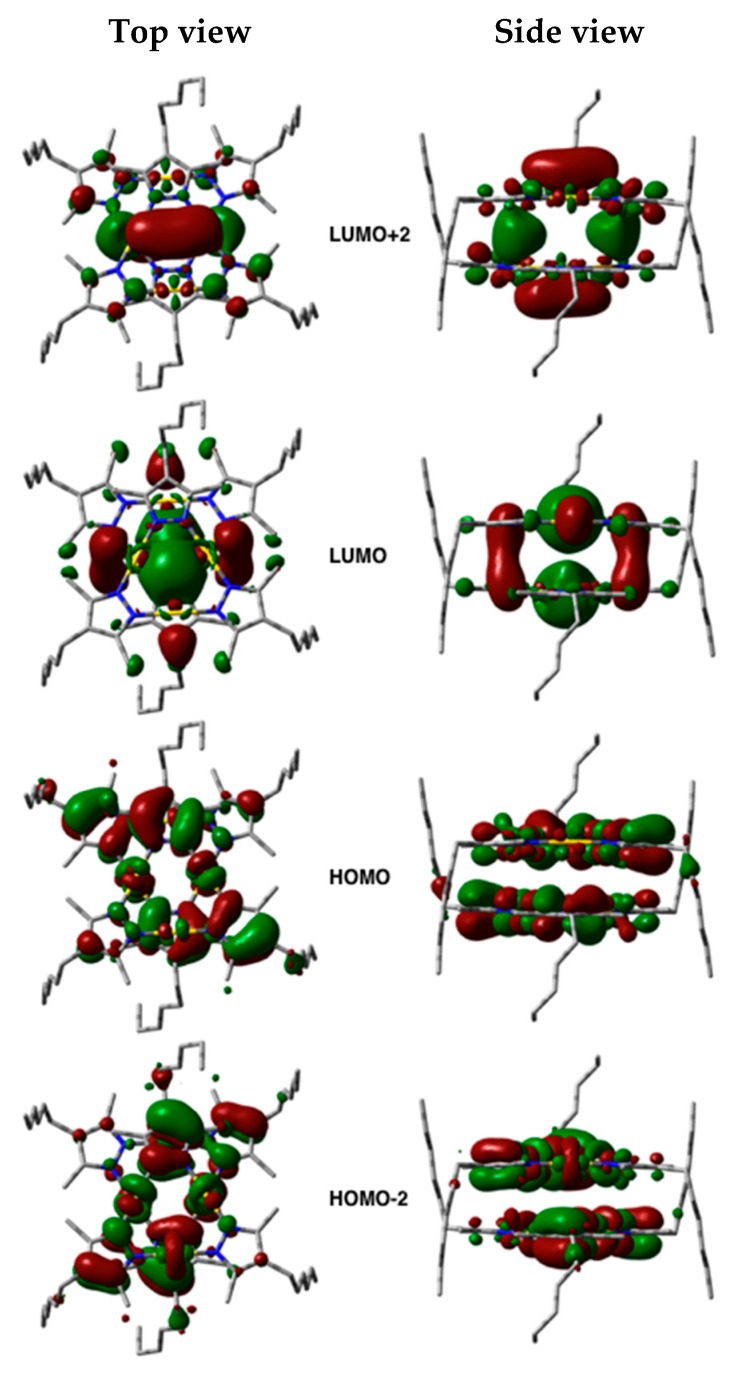
Molecular orbitals. of the **DT6** dimer obtained using DFT calculations employing B3LYP hybrid functionals with SDD (for Au atoms) and 6-311+G(d,p) (for the other atoms).

In order to clarify the luminescence mechanism in the crystal, the molecular orbitals, transition energies, and oscillator strengths (*f*) of the complexes were calculated using time-dependent density functional theory (TD-DFT) computations. The calculations were performed for the dimer model formed in the **DT6** crystal, as a representative example, using the initial conformation obtained from X-ray crystallography (Figure 2). The DFT calculations suggested that there are three allowed electron transitions are in the UV region; viz., HOMO → LUMO at 267.23 nm (*f* = 0.0002), HOMO–2 → LUMO at 262.04 nm (*f* = 0.0033), and HOMO → LUMO+2 at 255.46 nm (*f* = 0.0292) (see ESI). The calculations’ results were almost consistent with the excitation spectra of the complexes in the crystal (Figure 1b). As shown in Figure 3, the HOMO and HOMO–2 are delocalized mainly over the pyrazole ring, and the LUMO and LUMO+2 are localized mainly on a non-covalent aurophilic bonding between Au–Au atoms. Therefore, the results indicate that those electronic transitions in the excitation spectra can be assigned as a ligand-to-metal–metal charge transfer (LMMCT) transition. The broad photoluminescence band without vibronic structures observed at ~730 is consistent with the previously reported luminescence band for crystal of similar Au complexes [13,14], and it has been proposed that the luminescence is originated from metal-centered excimeric states based on the Au–Au interaction [14]. The computational results also support that the luminescence in the **DT*n*** crystals was emitted from the metal-centered excimeric states.

As shown in Figure 2c, Figure S4 and S5, in all the complexes, both Au–Au and Au–π interactions exist at two sites in each dimer. Additionally, both interactions are extended in **DT6**, as a result of which, the **DT6** dimers stack to form a column-like supramolecular polymer (Figure 2b). In contrast, these interactions are not extended in **DT7** and **DT8** crystals, and their dimers exist independently (Figure S4 and S5). We concluded that these multiple dual-mode intermolecular interactions forming the dimer and polymer were the cause of the highly efficient, in-air RTP observed in this system, which is likely enhanced due to the following three key effects: (i) The crystallinity of the complexes we prepared can prevent penetration of molecular oxygen from the air into the densely packed crystal lattice, as previously proposed by Tang et al. [36,37,38]. (ii) In the present system, the multiple intermolecular interactions can further restrict the internal motions in the crystal; therefore, *k*_nr_ is decreased more effectively, and the *Φ* of phosphorescence is increased, which is in line with the general working mechanism of AIE [1,2,3]. (iii) Each molecule in the developed system has three heavy Au atoms, which induce both internal and external heavy atom effects simultaneously. Consequently, both *k*_r_ and the rate constant for the spin-forbidden singlet-triplet intersystem crossing are effectively improved by the internal/external heavy atom effects. A combination of these effects likely brought about the very high *Φ* value in the in-air RTP. The multiple dual-mode intermolecular interactions observed in the crystals can increase the density of the molecular packing and can amplify all the aforementioned effects, resulting in highly efficient in-air RTP. Especially in **DT6,** the presence of extended, multiple dual-mode interactions would be expected to afford the strongest amplification, which was the case, and **DT6** showed the highest *Φ* value for the in-air RTP among the complexes prepared.

## 3. Materials and Methods

### 3.1. Preparation of Materials

The complete synthetic route for the preparation of the trinuclear Au(I) complexes (**DT*n***) is shown in Scheme S1. The complexes **DT*n*** were synthesized from the corresponding 4-alkyl-3,5-dimethylpyrazole ligand and (tht)AuCl, according to the procedure reported, with some modifications to the purification processes [32,33,34]. Unless otherwise noted, all solvents and reagents were purchased from commercial suppliers and were used without further purification. ^1^H NMR spectra were recorded using a ECS-400 spectrometer (JEOL, Tokyo, Japan) at 400 MHz using the residual proton in the NMR solvent as an internal reference. The complexes were fully characterized by high-resolution mass spectroscopy (HRMS), infrared spectroscopy (IR), and elemental analysis. Electrospray ionization mass spectra (ESI-MS) were measured using a JMS-T1000LC (JEOL, Tokyo, Japan). IR spectra were recorded using a FT/IR-4100 spectrometer (JASCO, Tokyo, Japan) as a KBr pellet. The melting points of the final products were determined as the peak onset in differential scanning calorimetry (DSC) with heating and cooling rates of 1.0 °C min^−1^.

**DT6**. 4-Hexyl-3,5-dimethylpyrazole (0.51 g, 2.8 mmol) and (tht)AuCl (1.0 g, 3.2 mmol) were dissolved in 30 mL of acetone. A 1.0 mol L^−1^ methanol solution (3.0 mL) of potassium hydroxide was added to the reaction mixture slowly (2 drops per second) with stirring. The solution was stirred for 2 h at room temperature, after which the white precipitate formed was collected by filtration. The crude product was purified on a silica gel column (eluent: CH_2_Cl_2_), and then recrystallized from a mixture of dichloromethane and acetone to give 0.69 g (0.61 mmol) of colorless needles (**DT6**) in 65% yield. mp 134 °C. ^1^H NMR (400 MHz, CDCl_3_, *δ*): 2.31 (t, *J* = 7.2 Hz; 6H; C*H_2_*(CH_2_)_4_CH_3_), 2.15 (s, 18H; pyrazole–C*H_3_),* 1.28–1.41 (m, 24H; CH_2_(C*H_2_*)_4_CH_3_), 0.88 (t, *J* = 6.8 Hz; 9H; (CH_2_)_5_C*H_3_*). ^13^C NMR (100 MHz, CDCl_3_, *δ*): 145.71 (3,5-C in pyrazole), 115.73 (4-C in pyrazole), 32.16 (pyrazole–*C*H_3_), 31.25 (–*C*H_2_(CH_2_)_4_CH_3_), 29.44 (CH_2_*C*H_2_(CH_2_)_3_CH_3_), 24.48 ((CH_2_)_2_*C*H_2_(CH_2_)_2_CH_3_), 23.08 ((CH2)_3_*C*H_2_CH_2_CH_3_), 14.50 ((CH_2_)_4_*C*H_2_CH_3_), 12.34((CH_2_)_5_*C*H_3_). FTIR (KBr): *ν* = 2956 cm^−1^ (C–H), 2922 cm^−1^ (C–H), 2852 cm^−1^ (C–H), 1515 cm^−1^ (C=C), 1456 cm^−1^ (C=C), 1429 cm^−1^ (C=N), 1371 cm^−1^ (C–H). Combustion elemental analysis calculated for C_33_H_57_Au_3_N_6_: C, 35.11; H, 5.09; N, 7.45; Au, 52.35. Found: C, 34.88; H, 4.94; N, 7.37; Ash, 48.4.

**DT7** and **DT8.** Compounds **DT7** and **DT8** were obtained by substituting 4-heptyl-3,5-dimethylpyrazole and 4-octyl-3,5-dimethylpyrazole for 4-hexyl-3,5-dimethylpyrazole in the above procedure, respectively. 

**DT7**: mp 117 °C. ^1^H NMR (400 MHz, CDCl_3_, *δ*): 2.31 (t, *J* = 7.5 Hz; 6H; C*H*_2_(CH_2_)_5_CH_3_), 2.13 (s, 18H; pyrazole–C*H*_3_), 1.28–1.43 (m, 30H; CH_2_(C*H*_2_)_5_CH_3_), 0.88 (t, *J* = 6.8 Hz; 9H; (CH_2_)_6_C*H*_3_). ^13^C NMR (100 MHz, CDCl_3_, *δ*): 145.51 (3,5-C in pyrazole), 115.89 (4-C in pyrazole), 32.29 (pyrazole-*C*H_3_), 31.26 (-*C*H_2_(CH_2_)_5_CH_3_), 29.72 (CH_2_*C*H_2_(CH_2_)_4_CH_3_), 29.60 ((CH_2_)_2_*C*H_2_(CH_2_)_3_CH_3_), 24.43 ((CH_2_)_3_*C*H_2_(CH_2_)_2_CH_3_), 23.05 ((CH_2_)_4_*C*H_2_CH_2_CH_3_), 14.50 ((CH_2_)_5_*C*H_2_CH_3_), 12.41 ((CH_2_)_6_*C*H_3_). FTIR (KBr): *ν* = 2955 cm^−1^ (C–H), 2922 cm^−1^ (C–H), 2852 cm^−1^ (C–H), 1515 cm^−1^ (C=C), 1465 cm^−1^ (C=C), 1452 cm^−1^ (C=C), 1436 cm^−1^ (C=N), 1374 cm^−1^ (C–H), 1357 cm^−1^ (C–H). Combustion elemental analysis calculated for C_36_H_63_Au_3_N_6_: C, 36.93; H, 5.42; N, 7.18; Au, 50.47. Found: C, 36.60; H, 5.38; N, 7.16; Ash, 36.6.

**DT8**: mp 114 °C. ^1^H NMR (400 MHz, CDCl_3_, *δ*): 2.30 (t, *J* = 7.5 Hz; 6H; C*H*_2_(CH_2_)_6_CH_3_), 2.11 (s, 18H; pyrazole–C*H*_3_), 1.27–1.41 (m, 36H; CH_2_(C*H*_2_)_6_CH_3_), 0.88 (t, *J* = 6.8 Hz; 9H; (CH_2_)_7_C*H*_3_). ^13^C NMR (100 MHz, CDCl_3_, *δ*): 145.51 (3,5-C in pyrazole), 115.58 (4-C in pyrazole), 32.29 (pyrazole-*C*H3), 31.30 (-*C*H_2_(CH_2_)_6_CH_3_), 29.92 (CH_2_*C*H_2_(CH_2_)_5_CH_3_), 29.80 ((CH_2_)_2_*C*H_2_(CH_2_)_4_CH_3_), 29.76 ((CH_2_)_3_*C*H_2_(CH_2_)_3_CH_3_), 24.52 ((CH_2_)_4_*C*H_2_(CH_2_)_2_CH_3_), 23.05 ((CH_2_)_5_*C*H_2_CH_2_CH_3_), 14.48 ((CH_2_)_6_*C*H_2_CH_3_), 12.30 ((CH_2_)_7_*C*H_3_). FTIR (KBr): *ν* = 2953 cm^−1^ (C–H), 2921 cm^−1^ (C–H), 2850 cm^−1^ (C–H), 1514 cm^−1^ (C=C), 1454 cm^−1^ (C=C), 1427 cm^−1^ (C=N), 1374 cm^−1^ (C–H), 1356 cm^−1^ (C–H). Combustion elemental analysis calculated for C_39_H_69_Au_3_N_6_: C, 38.62; H, 5.73; N, 6.93; Au, 48.72. Found: C, 38.45; H, 5.64; N, 6.90; Ash, 47.9.

### 3.2. Single Crystal X-ray Structure

The molecular structure and crystal packing structure were determined by single crystal X-ray structural analysis. Single crystals of gold(I) complexes were obtained by slow evaporation from a mixed solvent system (dichloromethane/acetone). Each crystal was mounted on a glass fiber, and the omega scanning technique was used to collect the reflection data using a D8 goniometer (Bruker, Billerica, MA, USA) with monochromatic Mo Kα radiation (*λ* = 0.71075 Å) for **DT6** or a automated four-circular-axis diffractometer AFC-5R (Rigaku, Tokyo, Japan) with graphite monochromatized Cu Kα radiation (*λ* = 1.54178 Å) for **DT7** and **DT8**. To investigate the actual crystal structures of the materials used, the measurements were performed at ambient temperature (296 K). For **DT6**, the initial structure of each unit cell was determined using a direct method in APEX3. The structural models were refined using a full-matrix least squares method in SHELXL-2014/6 [39,40]. All calculations were performed using SHELXL programs (University of Gottingen, Germany). For **DT7** and **DT8**, the initial structure in the unit cell was determined by a direct method using SIR92 [41]. The structure model was refined by full-matrix least-squares methods using SHELXL97 (University of Gottingen, Germany) [40]. All calculations were performed in the crystallographic software package WinGX (1.80, Farrugia, UK) [42]. When the alkyl chains were disordered, the occupancy of the atoms was separated into two parts. The crystal data are summarized in Table S1, and are included in the Cambridge Crystallographic Data Centre (CCDC) database as reference numbers CCDC 1910566–1910568 for **DT6**, **DT7**, and **DT8**, respectively. The indexed database contains additional supplementary crystallographic data for this paper and may be accessed without charge at http://www.ccdc.cam.ac.uk/conts/retrieving.html.

### 3.3. Photophysical Properties

UV–visible absorption and steady-state photoluminescence spectra were recorded using a V-550 absorption spectrophotometer (JASCO, Tokyo, Japan) and a F-7500 fluorescence spectrophotometer (Hitachi, Tokyo, Japan), respectively. The quantum yields of the photoluminescence were determined using a Quantaurus-QY absolute photoluminescence quantum yield spectrometer (C11347-01, Hamamatsu Photonics, Hamamatsu, Japan). Photoluminescence decay profiles were measured using a N_2_ laser (USHO Pulsed dye laser, KEC-160; wavelength, 337 nm; pulse width, 600 ps; 10 Hz) with a streak camera (C4334, Hamamatsu Photonics, Hamamatsu, Japan).

### 3.4. Computational Studies

The TD-DFT calculations were performed using the Gaussian 03 (revision E.01, Gaussian, Inc., Wallingford, CT, USA) program package, employing B3LYP hybrid functionals with SDD (for the Au atoms) and 6-311G+(d,p) (for the other atoms) basis sets [43]. The computation was carried out for the dimer formed in the crystal using the initial conformation obtained from the X-ray crystallography results. The vertical excitation energies and oscillator strengths were estimated for the 8 lowest transitions to excited singlets. 

## 4. Conclusions

In conclusion, crystalline trinuclear Au complexes were prepared and displayed highly efficient (*Φ* = ~75%) RTP in the presence of air. The complexes indicated multiple dual-mode intermolecular interactions (Au–Au and Au–π interactions), which led to the formation of a supramolecular dimer and a polymer, and these interactions played a crucial role in the highly efficient, in-air RTP. This intense RTP appeared in the long wavelength region (~730 nm), accompanied by an extremely large Stokes shift of 2.2 × 10^4^ cm^−1^ (450 nm). The efficient in-air RTP and the large Stokes shifts of the complexes developed are desirable properties which will be useful in development of photoluminescence materials. The Au complexes are expected to have potential applications in luminescent probes for bioimaging and for chemical sensing, and spectral conversion materials for displays, photovoltaic cells, plant cultivation, and related technologies [44,45,46,47,48,49].

## Figures and Tables

**Table 1:** Photophysical parameters of the crystalline complexes observed at room temperature in the presence of air.

Sample	*λ*_max_^lum^[nm] ^a)^	*Φ* ^a,b)^	*τ* [μs]	*k*_r_ [s^−1^] ^c)^	*k*_nr_ [s^−1^] ^c)^
DT6	733	0.748	13	5.8 × 10^4^	1.9 × 10^4^
DT7	726	0.609	13	4.7 × 10^4^	3.0 × 10^4^
DT8	733	0.625	12	5.2 × 10^4^	3.1 × 10^4^

^a)^ Excitation wavelength was 280 nm for steady-state measurements. ^b)^ The luminescence intensity was integrated from 350 nm to 875 nm for estimation of *Φ* (Figure S8). The measurements were performed in triplicate to check reproducibility, and the averaged value is indicated here. ^c)^ *k*_r_, the rate constant for radiative transition was estimated by *Φ*/*τ*, and *k*_nr_, the rate constant for non-radiative deactivation was estimated by (1 − *Φ*)/*τ*.

**Table 2:** Selected distances and angles between the closest neighboring molecules in room-temperature crystals of **DT*n***.

Sample	Au–Au Distance [Å]	Au–π Distance [Å]	*θ* [deg] ^a)^
**DT6**	3.40	3.58	15
**DT7**	3.29	3.38	2.4
**DT8**	3.25	3.39	7.0

^a)^ The angle, *θ*, was defined as the angle between the vector normal to the pyrazole ring and that passing through the centroid to the Au atom.

## Supplementary Materials

The following are available online at https://www.mdpi.com/1420-3049/24/24/4606/s1. Scheme S1: Synthesis route of **DT*n***; Figures S1–S3: ^1^H and ^13^C-NMR spectra; Figures S4 and S5: Crystal structures; Figure S6: Absorption spectra; Figure S7: Photoluminescence spectra in solution and in crystal; Figure S8: Photoluminescence spectra used for estimation of quantum yield; Figure S9: Decay profiles; Table S1: Crystallographic data; Table S2: Excitation energies and oscillator strengths.
