# Supplementary material for: Highly Efficient Aggregation-Induced Room-Temperature Phosphorescence with Extremely Large Stokes Shift Emitted from Trinuclear Gold(I) Complex Crystals
Source: Molecules. 2019 Dec 16;24(24):4606. doi: 10.3390/molecules24244606 (PMC6943708; doi:10.3390/molecules24244606)
Supplement: Supplementary file 1 [file molecules-24-04606-s001.pdf]

Supplementary Materials for:

Highly Efficient Aggregation-Induced Room-Temperature Phosphorescence with Extremely Large Stokes Shift Emitted from Trinuclear Gold(I) Complex Crystals

Osamu Tsutsumi, Masakazu Tamaru, Hitoya Nakasato, Shingo Shimai, Supattra Panthai, Yuki Kuroda, Kenta Yamaguchi, Kaori Fujisawa, and Kyohei Hisano*

Department of Applied Chemistry, Ritsumeikan University
1-1-1 Nojihigashi, Kusatsu 525-8577, Japan.
Email: tsutsumi@sk.ritsumei.ac.jp

Table of Contents

1. Preparation of Materials S2

2. Single Crystal X-Ray Structure Analysis S7

3. Photophysical properties of DT*n*. S10

4. TD-DFT calculations S13

1. Preparation of Materials.

The synthetic route for preparation of trinuclear Au(I) complexes (**DT*n***) is shown in Scheme S1.[1] Unless otherwise noted, all solvents and reagents were purchased from commercial suppliers and were used without further purification. ^{1}H NMR spectra were recorded on a JEOL ECS-400 spectrometer at 400 MHz using the residual proton in the NMR solvent as an internal reference. The complex **DT6** was first synthesized in this study, and were fully characterized by high-resolution mass spectroscopy (HRMS), infrared spectroscopy (IR), and elemental analysis. Electrospray ionization mass spectra (ESI-MS) were measured on JMS-T1000LC (JEOL). IR spectra were recorded on a JASCO FT/IR-4100 spectrometer using a KBr pellet. The melting points of the final products were determined at the peaks onset of differential scanning calorimetry (DSC) with heating and cooling rates at 1.0 °C/min.

Scheme S1. Synthetic route of **DT*n*** (*n* = 6–8)

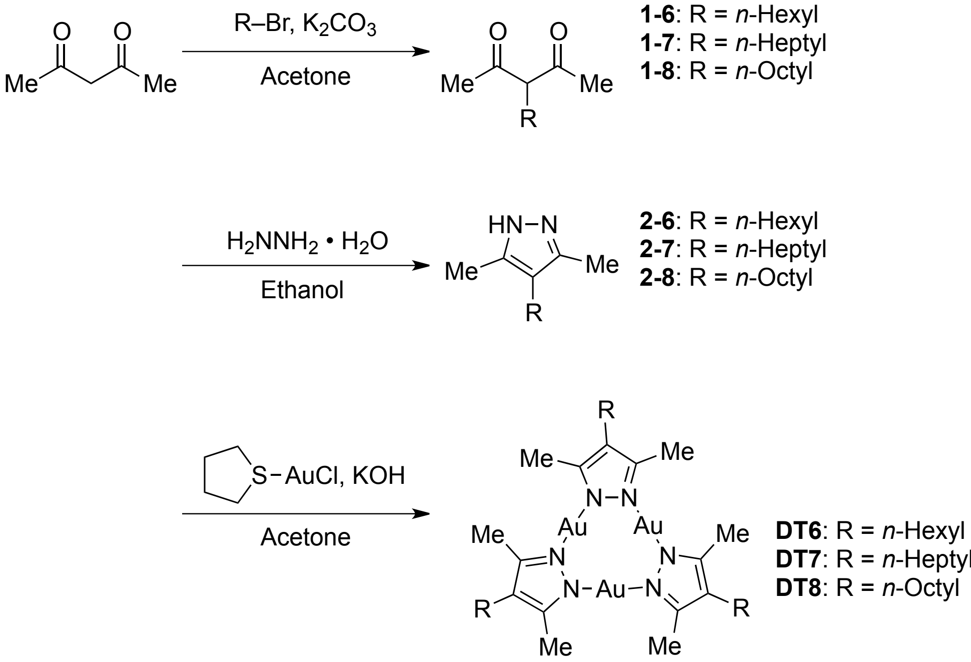

Synthesis of (tht)AuCl

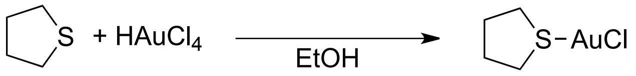

Tetrachloroauric(III) acid (1.0 g, 2.5 mmol) in 1.3 mL of water was added to 7.8 mL of ethanol and stirred at room temperature. To the resultant solution, 0.44 mL (4.9 mmol) of tetrahydrothiophene was added slowly, stirred at room temperature for 2 h, and then white precipitate was appeared. The precipitate was collected by filtration, washed with small amount of ethanol and air-dried to give 0.77 g (2.4 mmol) of white solid (**(tht)AuCl**) in 96% yield. ^{1}H NMR (400 MHz, $CDCl_3$, δ): 3.35–3.55 (br; 4H; S–C*H*$_2$CH$_2$), 2.13–2.31 (br; 4H; S–CH$_2$C*H*$_2$).

Synthesis of 1-*n*

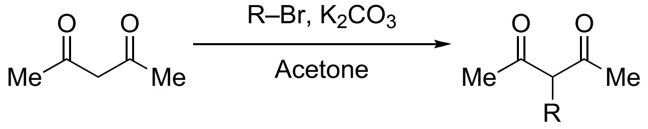

1-*n*

3-Hexyl-2,4-pentanedione (1-6). Acetyl acetone (2.0 mL, 20 mmol), 1-bromohexane (2.8 mL, 20 mmol), potassium carbonate (4.0 g, 29 mmol), and tetrabutylanmomium bromide (1.9 g, 5.9 mmol) were added to 30 mL of acetone, and the solution was stirred at room temperature for 22 h. A solid suspended in the reaction mixture was filtered off. After the filtrate was concentrated by evaporation, the product was dissolved in diethyl ether and washed with water (100 mL × 3 times), and brine (50 mL × 1 times). The organic layer was dried with sodium sulfate, and the solvent was removed to give 2.8 g (15 mmol) of pale-yellow oil (**1-6**) in 78% yield. ^{1}H NMR (400 MHz, $CDCl_3$, δ): 3.64 (t, J = 7.2 Hz; 1H; CH in pentadione), 2.17 (s, 6H; CH_3 in pentadione), 1.50–1.60 (m, 2H; $CH_2(CH_2)_4CH_3$), 1.15–1.39 (m, 8H; $CH_2(CH_2)_4CH_3$), 0.87 (t, J = 6.8 Hz; 3H; $CH_2(CH_2)_2CH_3$).

3-heptyl-2,4-pentanedione (1-7), and 3-octyl-2,4-pentanedione (1-8). According to above procedure, compounds **1-7** and **1-8** were obtained.

1-7: Yield = 50 %. ^{1}H NMR (400 MHz, $CDCl_3$, δ): 3.59 (t, J = 7.3 Hz; 1H; CH in pentadione), 2.16 (s, 6H; CH_3 in pentadione), 1.83 (t, J = 7.7 Hz; 2H; $CH_2(CH_2)_5CH_3$), 1.17–1.39 (m, 10H; $CH_2(CH_2)_5CH_3$), 0.86 (t, J = 6.8 Hz; 3H; $CH_2(CH_2)_5CH_3$).

1-8: Yield = 72 %. bp 117 °C/2 mmHg. ^{1}H NMR (400 MHz, $CDCl_3$, δ): 3.75 (t, J = 6.6 Hz; 1H; CH in pentadione), 2.17 (s, 6H; CH_3 in pentadione), 1.63–1.83 (m; 2H; $CH_2(CH_2)_6CH_3$), 1.27 (m, 12H; $CH_2(CH_2)_6CH_3$), 0.87 (t, J = 6.6 Hz; 3H; $CH_2(CH_2)_5CH_3$).

Synthesis of 2-*n*

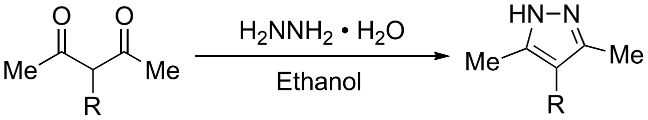

2-*n*

4-Hexyl-3,5-dimethylpyrazole (2-6). 3-Hexyl-2,4-pentanedione (**1-6**, 2.8 g, 15 mmol) was dissolved in 40 mL of ethanol. With stirring, 5.0 mL of hydrazine monohydrate (100 mmol) was added to the solution. After the reaction mixture was refluxed for 20 h, the mixture was diluted with 20 mL of water. Ethanol was removed by evaporation, and the solution was acidified (pH = 1) with hydrochloric acid (10%). The product was extracted with dichloromethane, washed with a saturated aqueous sodium hydroxycarbonate (50 mL) and brine (50 mL), and then the organic layer was dried with sodium sulfate. The solvent was removed completely under vacuum to give 1.6 g (8.9 mmol) of pale-yellow oil (**2-6**) in 59% yield. ^{1}H NMR (400 MHz, $CDCl_3$, δ): 8.94 (b; 1H; NH), 2.20 (s, 6H; CH_3 in pyrazole), 1.25–1.50 (m, 10H; $(CH_2)_5CH_3$), 0.88 (t, J = 6.8 Hz; 3H; $(CH_2)_5CH_3$).

4-Heptyl-3,5-dimethylpyrazole (2-7), and 4-octyl-3,5-dimethylpyrazole (2-7). According to above procedure, compounds **2-7** and **2-8** were obtained.

2-7: Yield = 89%. ^{1}H NMR (400 MHz, $CDCl_3$, δ): 2.41 (s, 6H; CH_3 in pyrazole), 1.20–1.60 (m, 12H; $(CH_2)_6CH_3$), 0.88 (t, J = 7.0 Hz; 3H; $(CH_2)_6CH_3$).

2-8: Yield = 45%. ^{1}H NMR (400 MHz, $CDCl_3$, δ): 2.19 (s, 6H; CH_3 in pyrazole), 1.23–1.57 (m, 14H; $(CH_2)_7CH_3$), 0.87 (t, J = 7.0 Hz; 3H; $(CH_2)_7CH_3$).

Synthesis of DT*n*

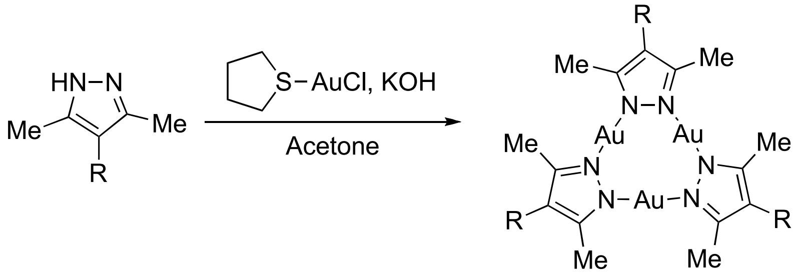

DT*n*

Tris(*μ*-3,5-dimethyl-4-hexylpyrazolato-*N,N'*)trigold(I) (DT6). 4-Hexyl-3,5-dimethylpyrazole (**2-6**, 0.51 g, 2.8 mmol) and 1.0 g of (tht)AuCl (3.2 mmol) was dissolved in 30 mL of acetone. With stirring, 1.0-mol L^{-1} methanol solution (3.0 mL) of potassium hydroxide was added into the reaction mixture slowly (2 drops s^{-1}). After the solution was stirred for 2 h at room temperature, the white precipitate formed was corrected by filtration. The crude product was purified on a silica gel column (eluent: CH_2Cl_2) and then recrystallized from a mixture of dichloromethane and acetone to give 0.69 g (0.61 mmol) of colorless needle (**DT6**) in 65% yield. mp 134 °C. ^{1}H NMR (400 MHz, $CDCl_3$, δ): 2.31 (t, J = 7.2 Hz; 6H; $CH_2(CH_2)_4CH_3$), 2.15 (s, 18H; pyrazole–CH_3), 1.28–1.41 (m, 24H; $CH_2(CH_2)_4CH_3$), 0.88 (t, J = 6.8 Hz; 9H; $(CH_2)_5CH_3$). ^{13}C NMR (100 MHz, $CDCl_3$, δ): 145.71 (3,5-C in pyrazole), 115.73 (4-C in pyrazole), 32.16 (pyrazole–CH_3), 31.25 (–$CH_2(CH_2)_4CH_3$), 29.44 ($CH_2CH_2(CH_2)_3CH_3$), 24.48 ($(CH_2)_2CH_2(CH_2)_2CH_3$), 23.08 ($(CH_2)_3CH_2CH_2CH_3$), 14.50 ($(CH_2)_4CH_2CH_3$), 12.34($(CH_2)_5CH_3$). FTIR (KBr): v = 2956 cm^{-1} (C–H), 2922 cm^{-1} (C–H), 2852 cm^{-1} (C–H), 1515 cm^{-1} (C=C), 1456 cm^{-1} (C=C), 1429 cm^{-1} (C=N), 1371 cm^{-1} (C–H). Anal calcd for $C_{33}H_{57}Au_3N_6$: C, 35.11; H, 5.09; N, 7.45; Au, 52.35. Found: C, 34.88; H, 4.94; N, 7.37; Ash, 48.4.

Tris(*μ*-3,5-dimethyl-4-heptylpyrazolato-*N,N'*)trigold(I) (DT7), and tris(*μ*-3,5-dimethyl-4-octylpyrazolato-*N,N'*)trigold(I) (DT8). According to above procedure, compounds **DT7** and **DT8** were obtained.

DT7: mp 117 °C. ^{1}H NMR (400 MHz, $CDCl_3$, δ): 2.31 (t, J = 7.5 Hz; 6H; $CH_2(CH_2)_5CH_3$), 2.13 (s, 18H; pyrazole–CH_3), 1.28–1.43 (m, 30H; $CH_2(CH_2)_5CH_3$), 0.88 (t, J = 6.8 Hz; 9H; $(CH_2)_6CH_3$). ^{13}C NMR (100 MHz, $CDCl_3$, δ): 145.51 (3,5-C in pyrazole), 115.89 (4-C in pyrazole), 32.29 (pyrazole-CH_3), 31.26 (-$CH_2(CH_2)_5CH_3$), 29.72 ($CH_2CH_2(CH_2)_4CH_3$), 29.60 ($(CH_2)_2CH_2(CH_2)_3CH_3$), 24.43 ($(CH_2)_3CH_2(CH_2)_2CH_3$), 23.05 ($(CH_2)_4CH_2CH_2CH_3$), 14.50 ($(CH_2)_5CH_2CH_3$), 12.41 ($(CH_2)_6CH_3$). FTIR (KBr): v = 2955 cm^{-1} (C–H), 2922 cm^{-1} (C–H), 2852 cm^{-1} (C–H), 1515 cm^{-1} (C=C), 1465 cm^{-1} (C=C), 1452 cm^{-1} (C=C), 1436 cm^{-1} (C=N), 1374 cm^{-1} (C–H), 1357 cm^{-1} (C–H). Anal. calcd. for $C_{36}H_{63}Au_3N_6$: C, 36.93; H, 5.42; N, 7.18; Au, 50.47. Found: C, 36.60; H, 5.38; N, 7.16; Ash, 36.6.

DT8: mp 114 °C. ^{1}H NMR (400 MHz, $CDCl_3$, δ): 2.30 (t, J = 7.5 Hz; 6H; $CH_2(CH_2)_6CH_3$), 2.11 (s, 18H; pyrazole–CH_3), 1.27–1.41 (m, 36H; $CH_2(CH_2)_6CH_3$), 0.88 (t, J = 6.8 Hz; 9H; $(CH_2)_7CH_3$). ^{13}C NMR (100 MHz, $CDCl_3$, δ): 145.51 (3,5-C in pyrazole), 115.58 (4-C in pyrazole), 32.29 (pyrazole-CH_3), 31.30 (-$CH_2(CH_2)_6CH_3$), 29.92 ($CH_2CH_2(CH_2)_5CH_3$), 29.80 ($(CH_2)_2CH_2(CH_2)_4CH_3$), 29.76 ($(CH_2)_3CH_2(CH_2)_3CH_3$), 24.52 ($(CH_2)_4CH_2(CH_2)_2CH_3$), 23.05 ($(CH_2)_5CH_2CH_2CH_3$), 14.48 ($(CH_2)_6CH_2CH_3$), 12.30 ($(CH_2)_7CH_3$). FTIR (KBr): v = 2953 cm^{-1} (C–H), 2921 cm^{-1} (C–H), 2850 cm^{-1} (C–H), 1514 cm^{-1} (C=C), 1454 cm^{-1} (C=C), 1427 cm^{-1} (C=N), 1374 cm^{-1} (C–H), 1356 cm^{-1} (C–H). Anal. calcd. for $C_{39}H_{69}Au_3N_6$: C, 38.62; H, 5.73; N, 6.93; Au, 48.72. Found: C, 38.45; H, 5.64; N, 6.90; Ash, 47.9.

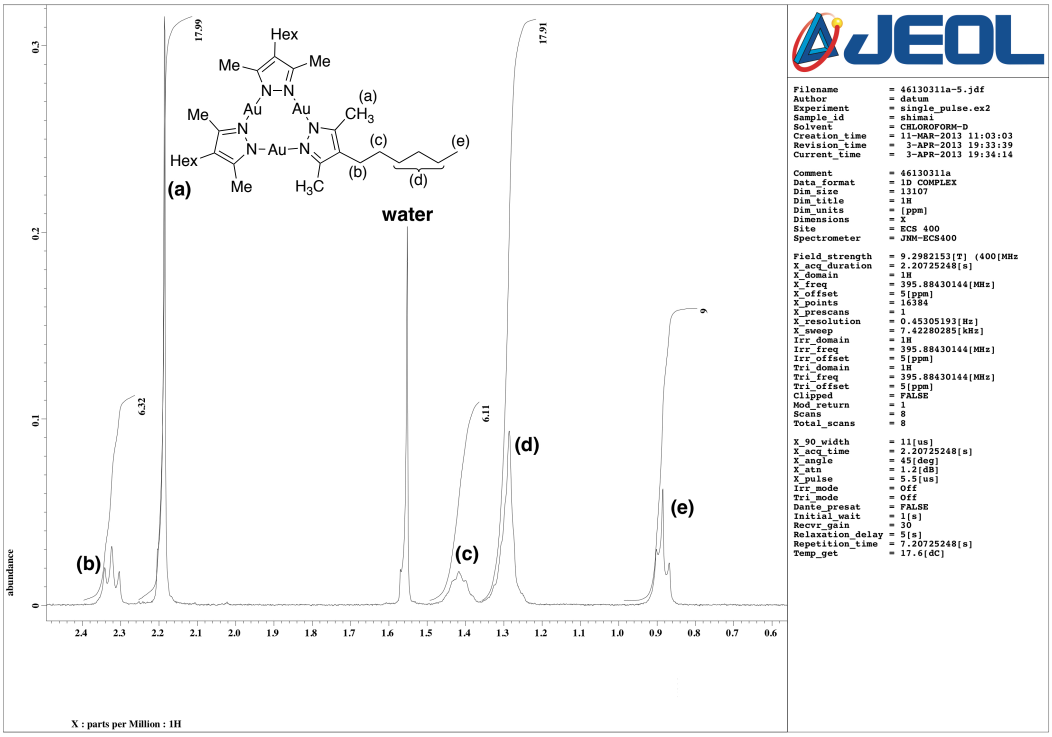

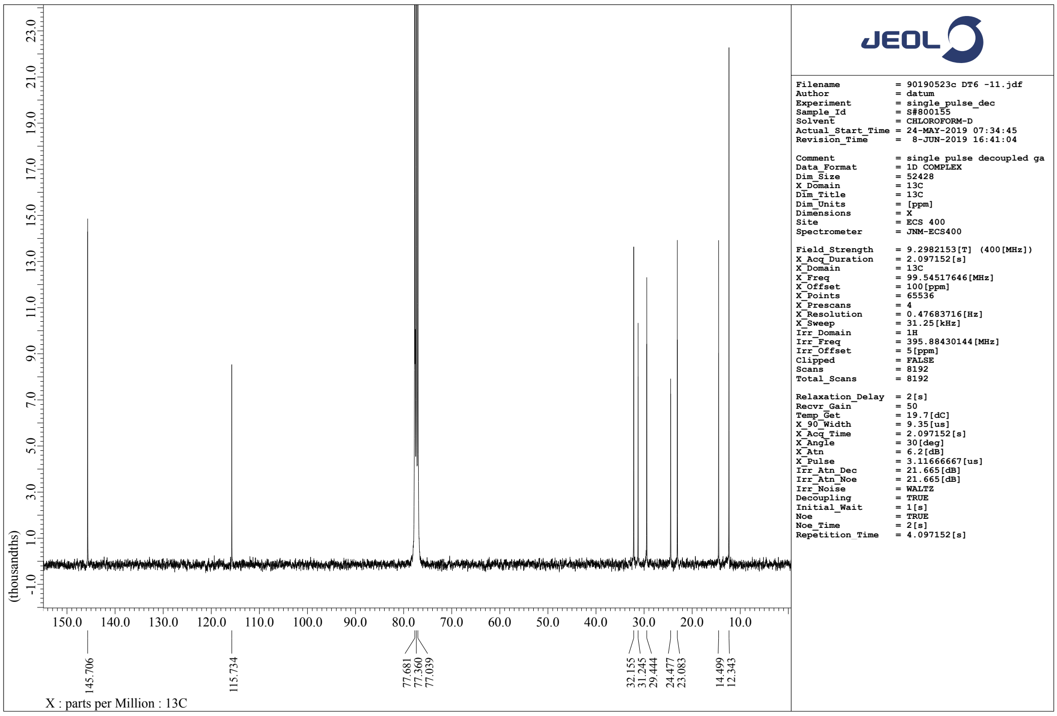

Figure S1. ^{1}H (top) and ^{13}C (bottom) NMR spectrum of **DT6** in $CDCl_3$.

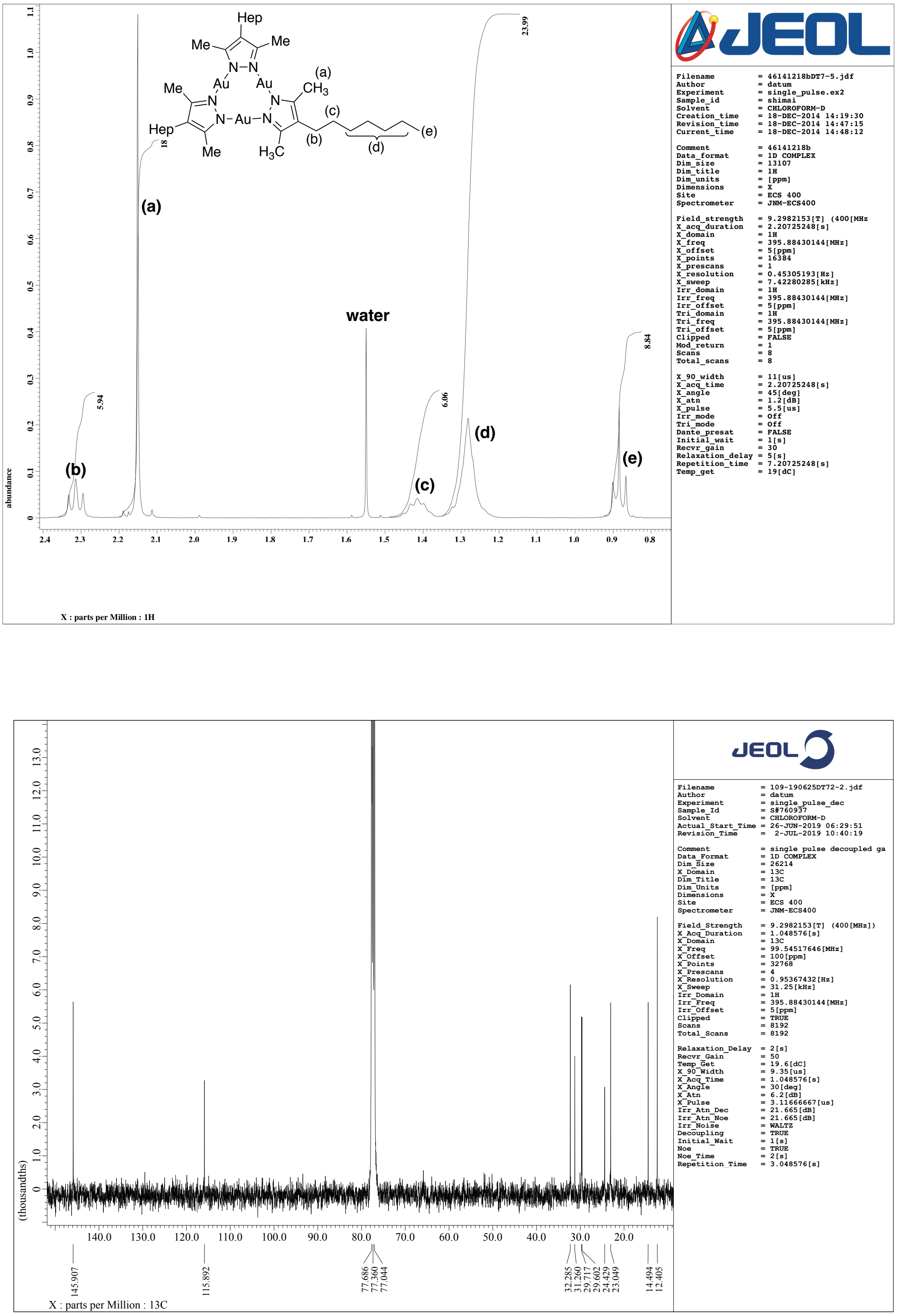

Figure S2. ^{1}H (top) and ^{13}C (bottom) NMR spectrum of **DT7** in $CDCl_3$.

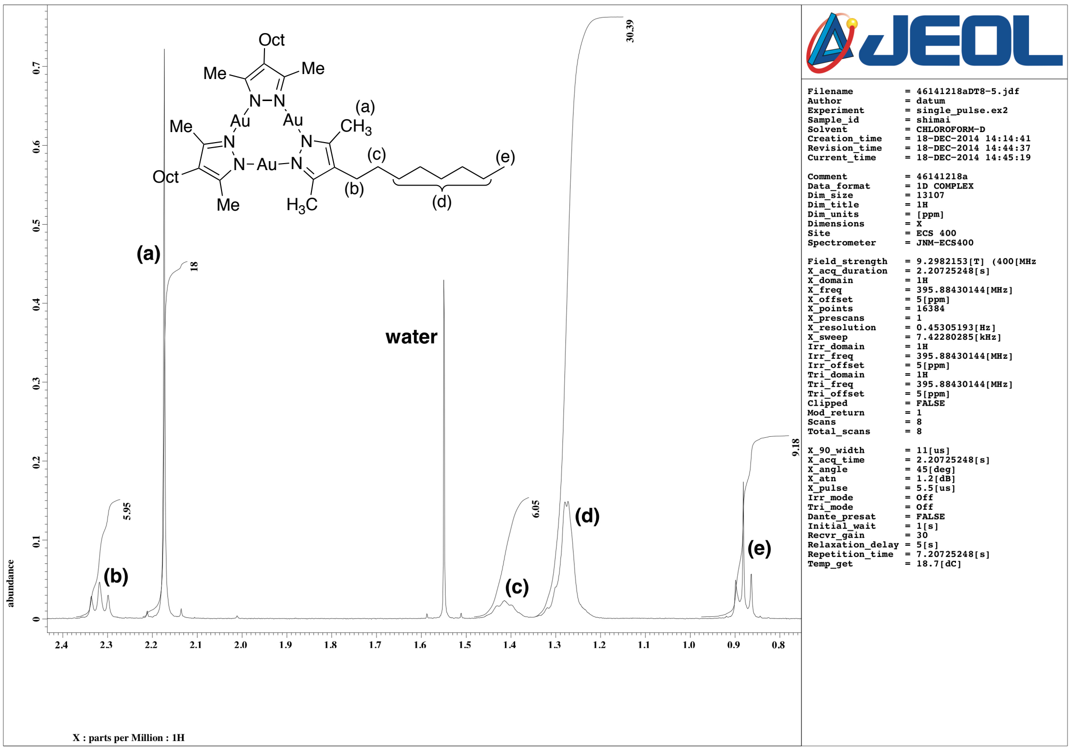

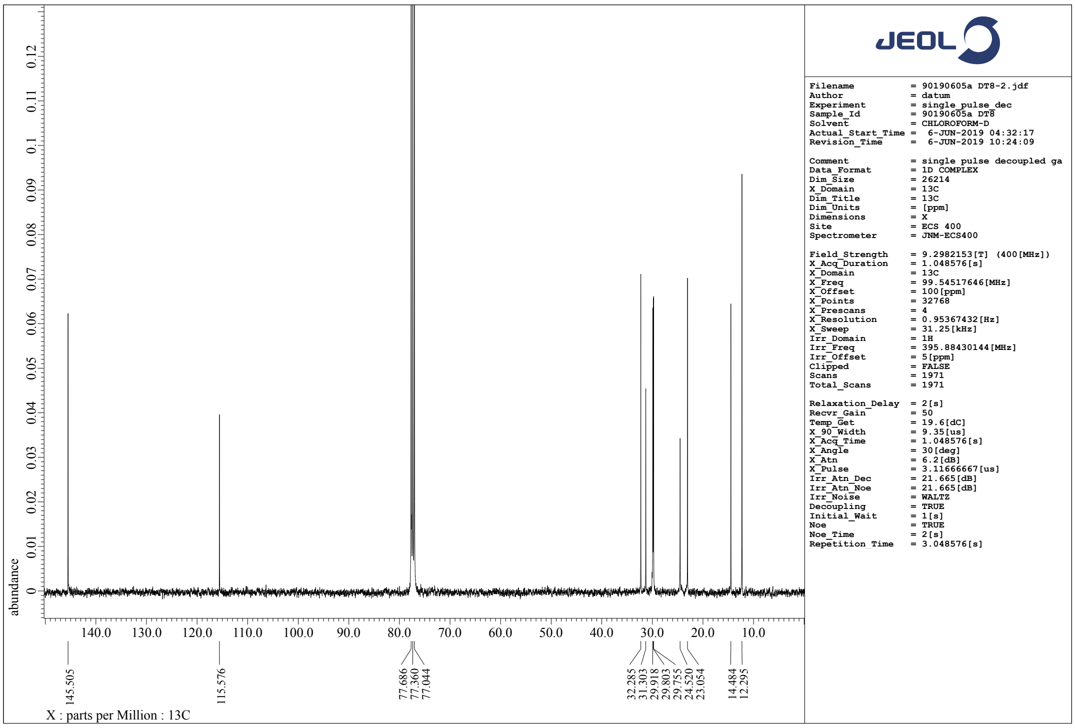

Figure S3. ^{1}H (top) and ^{13}C (bottom) NMR spectrum of **DT8** in $CDCl_3$.

2. Single Crystal X-Ray Structure Analysis.

The molecular structure and crystal packing structure were determined by single crystal X-ray structural analysis. Single crystals of Au(I) complexes were obtained by slow evaporation from a mixed solvent system (dichloromethane/acetone). Each crystal was mounted on a glass fiber and the omega scanning technique was used to collect the reflection data using a Bruker D8 goniometer with monochromatic Mo Kα radiation (λ = 0.71075 Å) for **DT6** or a Rigaku automated four-circular-axis diffractometer AFC-5R with graphite monochromatized Cu *K*α radiation (λ = 1.54178 Å) for **DT7** and **DT8**. To investigate the actual crystal structure of in-use materials, the measurements were performed at ambient temperature (296 K).

For **DT6**, the initial structure of each unit cell was determined using a direct method in APEX3. The structural models were refined using a full-matrix least squares method in SHELXL-2014/6.[2] All calculations were performed using SHELXL programs.

For **DT7** and **DT8**, the initial structure in the unit cell was determined by a direct method using SIR92.[3] The structure model was refined by full-matrix least-squares methods using SHELXL97.[2b] All calculations were performed on the crystallographic software package WinGX.[4]

The crystal data for complexes are summarized in Table S1. When alkyl chains were disordered, the occupancy of atoms was separated to two parts. The data in Table S1 have been indexed and are included in the Cambridge Crystallographic Data Center (CCDC) database with the following reference numbers of CCDC 1910566 for **DT6**, 1910567 for **DT7**, and 1910568 for **DT8**. The indexed database contains additional supplementary crystallographic data for this paper and may be accessed without charge at http://www.ccdc.cam.ac.uk/conts/retrieving.html. Complexes **DT7** and **DT8**, which have been already synthesized and characterized by Kim et al.,[1] showed the same structure as reported. The molecular structure and packing structure for **DT7** and **DT8** are shown in Figure S4 and S5.

Table S1 Crystallographic data of the **DT*n*** complexes obtained at room temperature.

Complex	**DT6**	**DT7**	**DT8**
Radiation type	Mo Kα	Cu Kα	Cu Kα
Wavelength (Å)	0.71073	1.54178	1.54178
Empirical formula	$C_{33}H_{57}Au_3N_6$	$C_{36}H_{63}Au_3N_6$	$C_{39}H_{69}Au_3N_6$
Formula weight	1128.74	1170.84	1212.90
Temperature (K)	296	296	296
Crystal habit	block	block	block
Crystal color	colorless	colorless	colorless
Crystal size (mm)	0.10 × 0.10 × 0.10	0.26 × 0.09 × 0.06	0.61 × 0.52 × 0.13
Crystal system	Monoclinic	Triclinic	Triclinic
Space group	*P 1 21/n 1*	*P -1*	*P -1*
a (Å)	17.8775(6)	10.8827(18)	11.382(3)
b (Å)	8.2352(2)	11.9567(13)	11.633(3)
c (Å)	26.2483(8)	16.9742(14)	18.685(4)
α (deg)	90.00	88.144(9)	82.52(2)
β (deg)	91.0320(10)	89.116(12)	81.34(2)
γ (deg)	90.00	69.189(10)	66.543(18)
V (Å^3)	3863.8(2)	2063.5(4)	2236.9(10)
Z	4	2	2
R_{int}	0.042	0.043	0.023
$R\ [F^2 \rightarrow 2\sigma\ (F^2)]$	0.0413	0.0492	0.0614
$wR\ (F^2)$	0.1657	0.1447	0.1916
S	1.047	1.031	1.044

(a)

(b)

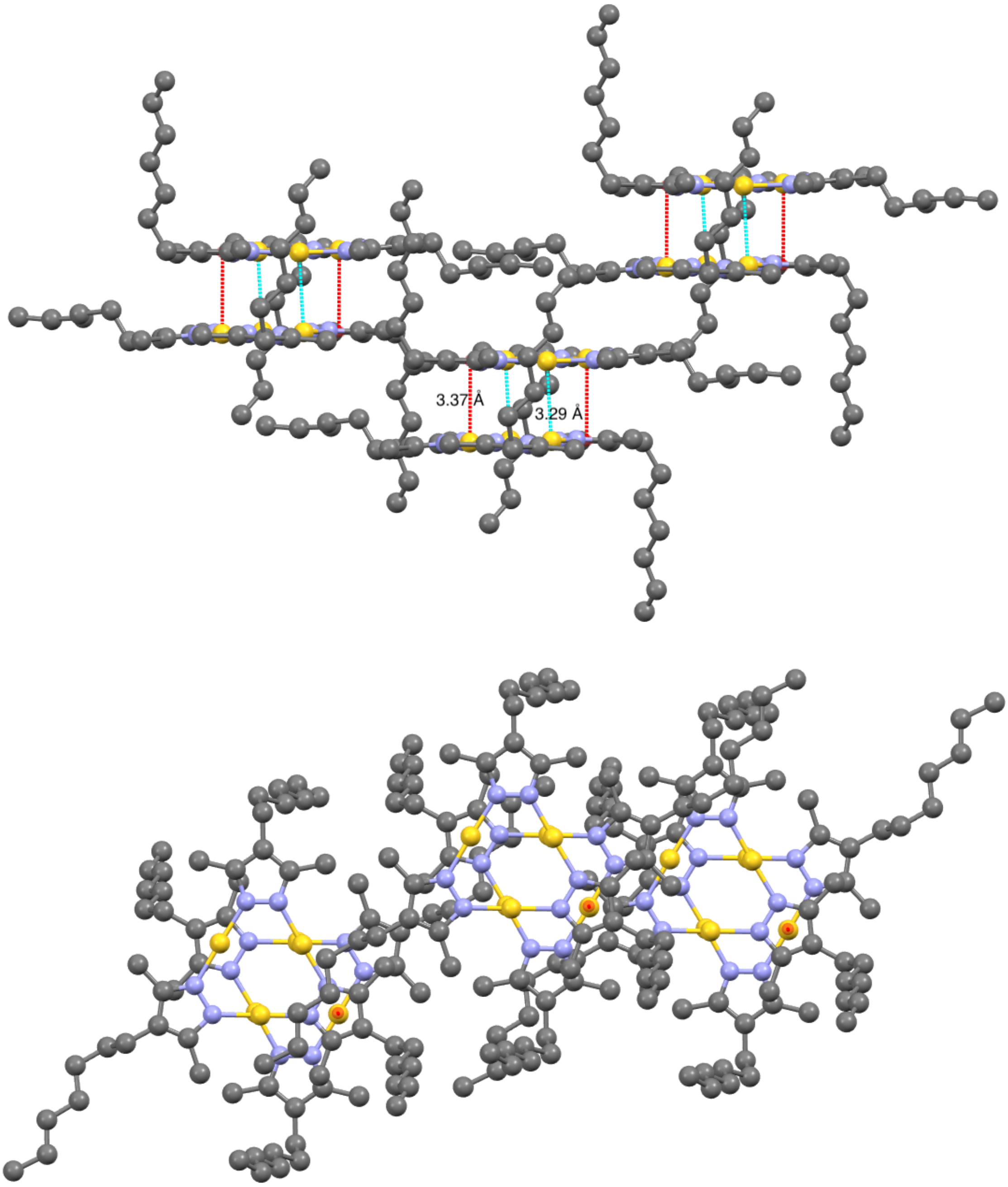

Figure S4. Crystal structure of **DT7** at room temperature. Three dimers are extracted and indicated in the figure. (a) side view, (b) top view. For clarity, H atoms are omitted, and only the major components are shown. Atom color legend: grey, C; purple, N; yellow, Au; red, centroid of pyrazole ring. Intermolecular Au–Au and Au–π interactions are indicated using light blue and red lines, respectively.

(a) (b)

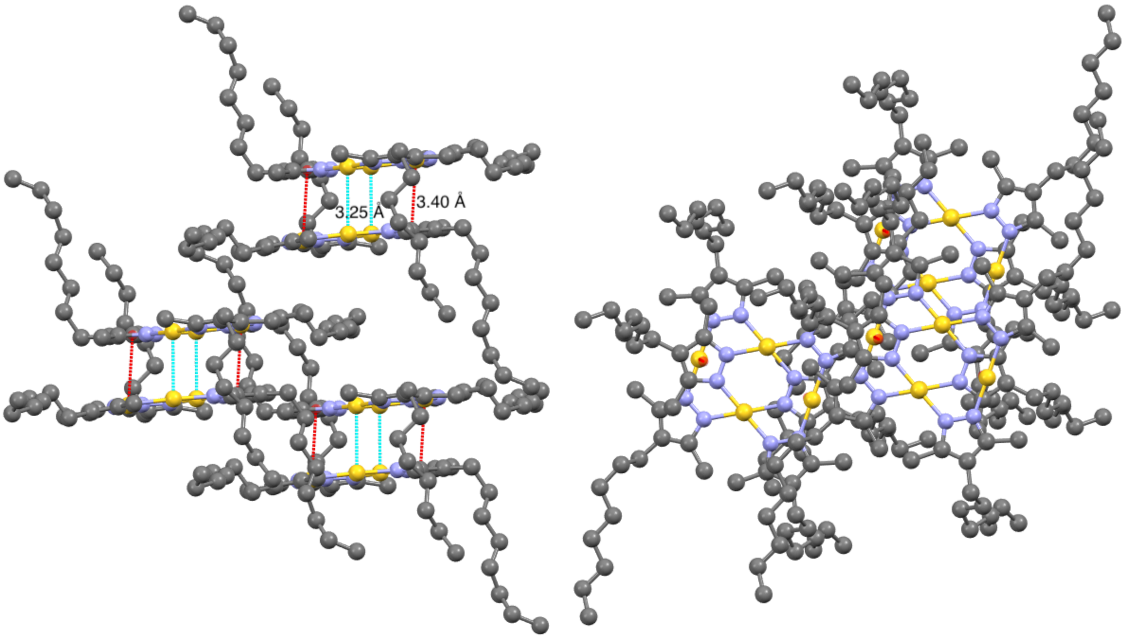

Figure S5. Crystal structure of **DT8** at room temperature. Three dimers are extracted and indicated in the figure. (a) side view, (b) top view. For clarity, H atoms are omitted, and only the major components are shown. Atom color legend: grey, C; purple, N; yellow, Au; red, centroid of pyrazole ring. Intermolecular Au–Au and Au–π interactions are indicated using light blue and red lines, respectively.

3. Photophysical properties of DT*n*.

UV-visible absorption and steady-state photoluminescence spectra were recorded on a JASCO V-550 absorption spectrophotometer and on a Hitachi F-7500 fluorescence spectrophotometer, respectively. Quantum yields of photoluminescence were determined using a Quantaurus-QY absolute photoluminescence quantum yield spectrometer (C11347-01, Hamamatsu). Photoluminescence decay profiles were measured using a N_2 laser (USHO Pulsed dye laser, KEC-160; wavelength 337 nm; pulse width 600 ps; 10 Hz) with a streak camera (Hamamatsu, C4334).

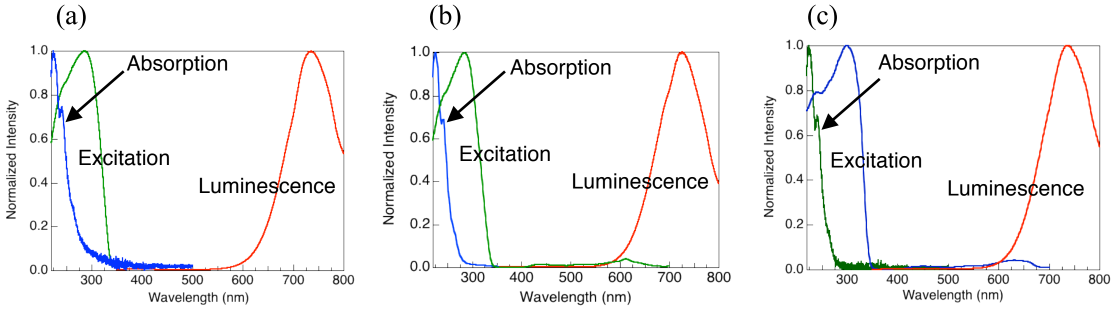

Figure S6. Absorption spectra in dilute hexane solution (10^{-5} mol L^{-1}), photoluminescence spectra in crystal, and excitation spectra in crystal of **DT*n***. The excitation wavelength was 280 nm for the luminescence spectra, and the luminescence was monitored at their luminescence maxima for the excitation spectra. (a) **DT6**, (b) **DT7**, (c) **DT8**.

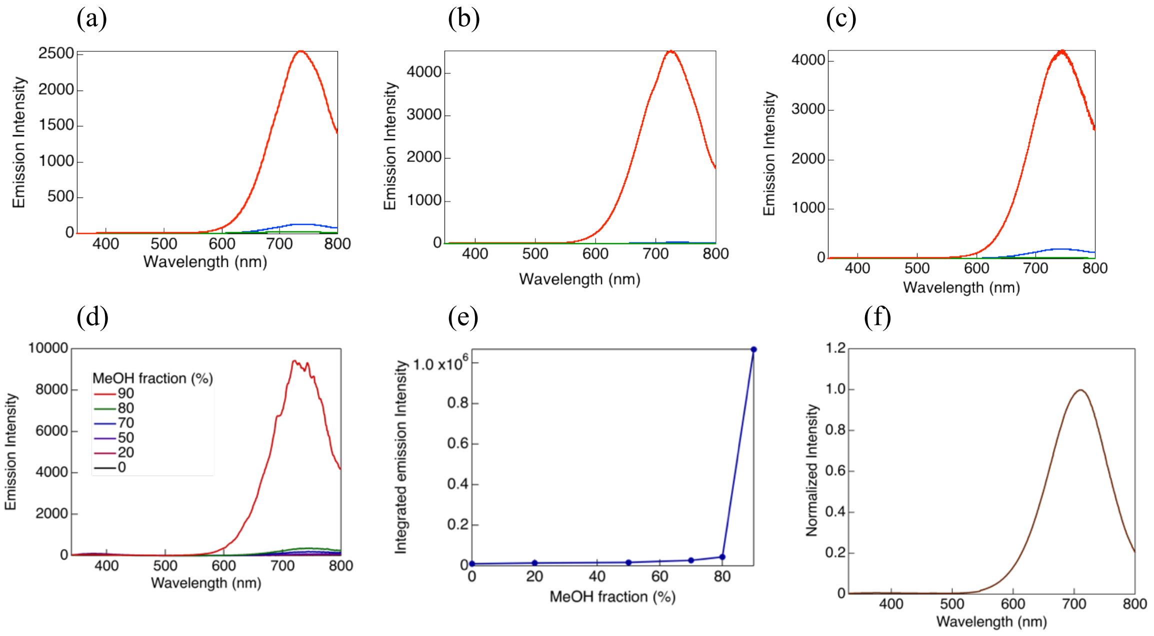

Figure S7. Comparison of photoluminescence spectra in hexane solution with that in crystal. Excitation wavelength was 280 nm for all measurements. (a) **DT6** (green, 6 x 10^{-5}-mol L^{-1} solution; blue, 3 x 10^{-3}-mol L^{-1} solution; red, crystal), (b) **DT7** (green, 5 x 10^{-5}-mol L^{-1} solution; blue, 3 x 10^{-3}-mol L^{-1} solution; red, crystal), (c) **DT8** (green, 5 x 10^{-6}-mol L^{-1} solution; blue, 1 x 10^{-2}-mol L^{-1} solution; red, crystal). (d) Photoluminescence spectra of **DT6** in the CH_2Cl_2/methanol mixed solvents with different methanol concentrations ([**DT6**] = 1.0×10^{-4} mol L^{-1}, λ_{ex} = 280 nm) in the ambient condition: the methanol fractions (vol%) in the solvent are indicated in the figure. (e) Integrated emission intensity of **DT6** in the CH_2Cl_2/methanol mixture as a function of methanol fractions (vol%): the luminescence spectra were integrated in the wavelength range from 500 nm to 800 nm. (f) Photoluminescence spectra of **DT6** crystal in nitrogen atmosphere at 0 °C. Excitation wavelength was 280 nm.

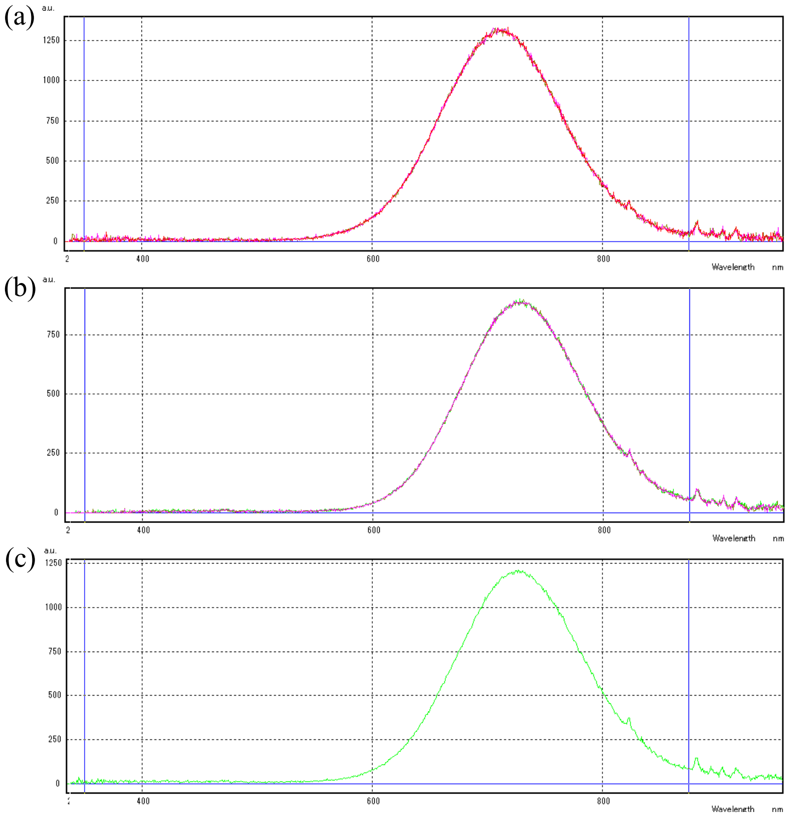

Figure S8. Photoluminescence spectra used for estimation of quantum yield of room-temperature phosphorescence in air. Excitation wavelength was 280 nm. The luminescence intensity was integrated from 350 nm to 875 nm for estimation of the quantum yield. The measurements were performed three times to check reproducibility. (a) **DT6**, (b) **DT7**, (c) **DT8**.

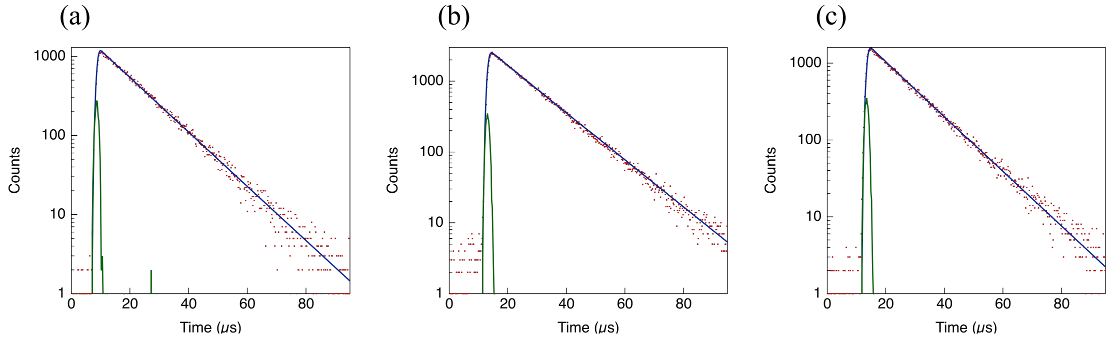

Figure S9. Decay profiles for room-temperature phosphorescence in crystals of **DT*n***: red, observed luminescence decay; blue, fitting curve; green, instrument response function. (a) **DT6**, (b) **DT7**, (c) **DT8**.

4. TD-DFT calculations

The TD-DFT calculations were performed for **DT6** as a representative example using the Gaussian 03 (revision E.01) program package, employing B3LYP hybrid functionals with SDD (for the Au atoms) and 6-311+G (d,p) (for the other atoms) basis sets.[5] The single point energy calculation was carried out for the dimer of **DT6** formed in the crystal using the conformation obtained from the X-ray crystallography results. The vertical excitation energies and oscillator strengths were estimated for the 8 lowest transitions to excited singlets and are listed below.

Table S2 List of excitation energies and oscillator strengths of **DT6**

Excited State 1:	Singlet-A	4.6396 eV	267.23 nm	*f* = 0.0002
354[†] → 355[†]	0.64686			
354 → 357	-0.17800			
Excited State 2:	Singlet-A	4.6410 eV	267.15 nm	*f* = 0.0000
353 → 355	0.64473			
353 → 357	-0.22052			
Excited State 3:	Singlet-A	4.7294 eV	262.16 nm	*f* = 0.0000
351 → 355	0.61017			
351 → 357	-0.18303			
354 → 356	0.14935			
354 → 363	-0.11942			
Excited State 4:	Singlet-A	4.7315 eV	262.04 nm	*f* = 0.0033
352 → 355	0.65030			
352 → 357	-0.18620			
Excited State 5:	Singlet-A	4.7735 eV	259.73 nm	*f* = 0.0000
350 → 355	0.63747			
350 → 357	-0.16392			
Excited State 6:	Singlet-A	4.7786 eV	259.46 nm	*f* = 0.0000
349 → 355	-0.19682			
352 → 358	-0.19123			
352 → 361	-0.16347			
353 → 355	0.12087			
353 → 357	0.41895			
353 → 360	-0.20240			
354 → 356	0.15435			
354 → 358	0.18503			
354 → 359	0.10208			
354 → 361	0.14851			
Excited State 7:	Singlet-A	4.8534 eV	255.46 nm	*f* = 0.0292
350 → 358	-0.16490			
350 → 361	-0.11772			
351 → 358	-0.11966			
353 → 356	-0.12419			
353 → 359	0.18410			
354 → 357	0.51576			
354 → 360	-0.22192			

Excited State 8:	Singlet-A	4.8914 eV	253.47 nm	*f* =	0.0000
348 → 356	0.12803				
349 → 355	0.48082				
351 → 357	-0.12769				
352 → 358	-0.10484				
353 → 357	0.18130				
354 → 356	-0.32182				
354 → 359	0.15845				

† Orbital numbers 354 and 355 correspond to HOMO and LUMO, respectively.

References and notes

[1] (a) J. Barberá, A. Elduque, R. Giménez, L. A. Oro, J. L. Serrano, *Angew. Chem. Int. Ed. Engl.* **1996**, *35*, 2832. (b) S. J. Kim, S. H. Kang, K. M. Park, H. Kim, W. C. Zin, M. G. Choi, K. Kim, *Chem. Mater.* **1998**, *10*, 1889. (c) J. Cored, O. Crespo, J. L. Serrano, A. Elduque, R. Giménez, *Inorg. Chem.* **2018**, *57*, 12632.

[2] (a) G. M. Sheldrick, *SHELXS-2014, Program for Crystal Structure Solution*, University of Göttingen, 2014; (b) G. M. Sheldrick, *Acta Crystallogr., Sect. A* **2008**, *64*, 112.

[3] A. Altomare, G. Cascarano, C. Giacovazzo, A. Guagliardi, *J. Appl. Crystallogr.* **1993**, *26*, 343.

[4] L. J. Farrugia, *J. Appl. Crystallogr.* **1999**, *32*, 837.

[5] M. J. Frisch, G. W. Trucks, H. B. Schlegel, G. E. Scuseria, M. A. Robb, J. R. Cheeseman, J. A. Montgomery, Jr., T. Vreven, K. N. Kudin, J. C. Burant, J. M. Millam, S. S. Iyengar, J. Tomasi, V. Barone, B. Mennucci, M. Cossi, G. Scalmani, N. Rega, G. A. Petersson, H. Nakatsuji, M. Hada, M. Ehara, K. Toyota, R. Fukuda, J. Hasegawa, M. Ishida, T. Nakajima, Y. Honda, O. Kitao, H. Nakai, M. Klene, X. Li, J. E. Knox, H. P. Hratchian, J. B. Cross, V. Bakken, C. Adamo, J. Jaramillo, R. Gomperts, R. E. Stratmann, O. Yazyev, A. J. Austin, R. Cammi, C. Pomelli, J. W. Ochterski, P. Y. Ayala, K. Morokuma, G. A. Voth, P. Salvador, J. J. Dannenberg, V. G. Zakrzewski, S. Dapprich, A. D. Daniels, M. C. Strain, O. Farkas, D. K. Malick, A. D. Rabuck, K. Raghavachari, J. B. Foresman, J. V. Ortiz, Q. Cui, A. G. Baboul, S. Clifford, J. Cioslowski, B. B. Stefanov, G. Liu, A. Liashenko, P. Piskorz, I. Komaromi, R. L. Martin, D. J. Fox, T. Keith, M. A. Al-Laham, C. Y. Peng, A. Nanayakkara, M. Challacombe, P. M. W. Gill, B. Johnson, W. Chen, M. W. Wong, C. Gonzalez, and J. A. Pople, Gaussian 03, Revision E.01, Gaussian, Inc., Wallingford, CT, USA **2004**.
